# Time from Injury to Initial Operation May Be the Sole Risk Factor for Postoperative Leakage in AAST-OIS 2 and 3 Traumatic Duodenal Injury: A Retrospective Cohort Study

**DOI:** 10.3390/medicina58060801

**Published:** 2022-06-14

**Authors:** Yun Chul Park, Hyo Sin Kim, Do Wan Kim, Wu Seong Kang, Young Goun Jo, Hyunseok Jang, Euisung Jeong, Naa Lee

**Affiliations:** 1 Division of Trauma, Department of Surgery, Chonnam National University Medical School and Hospital, Chonnam National University, Gwangju 61469, Korea; kontikithor@gmail.com (Y.C.P.); thinkjo82@gmail.com (Y.G.J.); bluetholic@naver.com (H.J.); bleocrj@hanmail.net (E.J.); tr00013@naver.com (N.L.); 2 Department of Surgery, Chonnam National University Medical School and Hospital, Chonnam National University, Gwangju 61469, Korea; gideon0504@gmail.com; 3 Department of Thoracic and Cardiovascular Surgery, Chonnam National University Hospital, Chonnam National University Medical School, Gwangju 61469, Korea; maskjoa@naver.com; 4 Department of Trauma Surgery, Jeju Regional Trauma Center, Cheju Halla General Hospital, Jeju 63127, Korea

**Keywords:** duodenal trauma, primary repair, pyloric exclusion, postoperative leakage

## Abstract

*Background and Objectives*: Traumatic duodenal injury is a rare disease with limited evidence. We aimed to evaluate the risk factors for postoperative leakage and outcomes of pyloric exclusion after duodenal grade 2 and 3 injury. *Materials and Methods*: We reviewed a prospectively collected trauma database for the period January 2004–December 2020. Patients with grade 2 and 3 traumatic duodenal injury were included. To identify the risk factors for postoperative leakage, we used a stepwise multivariable logistic regression model and a least absolute shrinkage and selection operator (LASSO) logistic model. We constructed a receiver operator characteristic (ROC) curve to predict risk factors for postoperative leakage. *Results*: During the 17-year period, 179,887 trauma patients were admitted to a regional trauma center in Korea. Of these patients, 74 (0.04%) had duodenal injuries. A total of 49 consecutive patients had grade 2 and 3 traumatic duodenal injuries and underwent laparotomy. The incidence of postoperative leakage was 32.6% (16/49). Overall mortality was 18.4% (9/49). A stepwise multivariable logistic regression and LASSO logistic regression model showed that time from injury to initial operation was the sole statistically significant risk factor. The ROC curve at the optimal threshold of 15.77 h showed the following: area under ROC curve, 0.782; sensitivity, 68.8%; specificity, 87.9%; positive predictive value, 73.3%; and negative predictive value, 85.3%. There was no significant difference in outcomes between primary repair alone and pyloric exclusion. *Conclusions*: Time from injury to initial operation may be the sole significant risk factor for postoperative duodenal leakage. Pyloric exclusion may not be able to prevent postoperative leakage.

## 1. Introduction

To date, the incidence of traumatic duodenal injury was reported to be rare, ranging from 3% to 5% [1]. The duodenum, similar to the pancreas, is located in the central abdomen and is covered by other internal organs, which protects it from external forces of trauma. Thus, many trauma surgeons have limited experience of surgery for duodenal trauma. Moreover, there has been limited high quality evidence of such trauma, including that of large cohorts or prospective data [2]. The majority of recommendations for traumatic duodenal injury by the World Society of Emergency Surgery (WSES) and the American Association for the Surgery for Trauma (AAST) is weak, and the supporting evidence provided in terms of operative management of duodenal perforation is moderate to low [2]; the American Association for Surgery of Trauma Organ Injury Scaling (AAST-OIS) for traumatic duodenal injury is between 2 and 4. The only strong recommendation is an emergency operation for unstable patients [2]. The guidelines recommend that primary repair should be considered regardless of grade of injury, and pyloric exclusion may be considered for grade 3 duodenal or high-grade injury [2]. However, the optimal indications for primary repair and other surgical strategies, including pyloric exclusion, remain controversial. In a recent nationwide analysis of types of duodenal injury repair using the National Trauma Databank (NTDB), Aiolfi et al. [3] reported 78.5% primary repair, 19.2% pyloric exclusion, 19.1% duodenojejunostomy, and 3.4% pancreaticojejunostomy in the period from 2002 to 2014. Notably, according to NTDB data, the use of primary repair has increased [3]. In the modern era, trends may be shifting toward a less invasive procedure.

One of the most crucial factors in determining surgical strategy is postoperative leakage, which can induce serious complications, such as surgical site infection, sepsis, malnutrition, and death. However, risk factors for postoperative leakage for duodenal trauma remain unclear. The trauma surgeon’s decision whether to use primary repair alone or another surgical option is crucial. To date, several risk factors, such as delayed operation, were mentioned in the literature [4,5]; however, the evidence is limited.

In this study, we aimed to identify the risk factors for postoperative leakage after AAST-OIS 2 or 3 duodenal injury repair surgery. The primary outcome of our study was postoperative leakage, and the secondary outcome was the efficacy of pyloric exclusion.

## 2. Materials and Methods

### 2.1. Study Population

The present study was approved by the institutional review board of our institution (CNUH-2021-159). We collected and reviewed data from a prospectively collected trauma database, for the period between January 2004 and December 2020, at a tertiary referral center (Chonnam National University Hospital), which was nominated as a regional trauma center in South Korea in 2013. This regional trauma center in South Korea corresponds to a level 1 trauma center in the United States. Our 1800-bed teaching hospital serves a population of 3 million people, and more than 500 patients with major trauma [(injury severity score (ISS) > 15) are admitted annually. We searched for trauma patients who underwent laparotomy due to grade 2 or 3 traumatic duodenal injury. Injury grade was evaluated according to AAST-OIS [6]. The exclusion criteria were as follows: (1) patients who died by postoperative day 7 because of the inability to detect postoperative leakage in this short period (*n* = 2); (2) patients with grade 1 duodenal injury that was managed via conservative treatment (*n* = 14); (3) patients with grade 4 or grade 5 duodenal injury that could not be closed via primary repair only due to massive destruction of the duodenum or an adjacent organ (*n* = 7); and (4) patients who underwent pancreaticoduodenectomy (*n* = 1).

### 2.2. Clinical Variables and Definitions

The patients’ demographic and clinical data including age, gender, mechanism of injury (blunt or penetrating), ISS, Glasgow Coma Scale (GCS) on admission, vasopressor use in emergency department, time at the scene, time from injury to the hospital, time from injury to the operating room, time from injury to initial operation (skin incision time), operative data, length of stay, transfusion, and mortality and morbidity outcomes were collected and analyzed. The Charlson Comorbidity score was calculated using age and other morbidities [7].

The severity of duodenal injury was scored using the AAST-OIS [6]. Postoperative leakage was defined as the following: (1) bile containing fluid leakage via a drain catheter, which was confirmed by abdominal computed tomography (CT) or fistulography, and (2) leakage that was confirmed intraoperatively during reoperation due to peritonitis or intra-abdominal abscess. Pyloric exclusion consists of gastrotomy, pyloric exclusion, and gastrojejunostomy [8]. The decision to perform pyloric exclusion was taken by the operator based on the status of the injured duodenum, degree of contamination, or the patient’s instability. All complications after surgery were classified according to the recommendations by Dindo et al. [9]. Intra-abdominal complication was defined as a complication confined to the abdomen, such as wound infection, ileus, or intra-abdominal abscess. Systemic complication was defined as other systemic complications, such as pneumoniae or acute renal injury. The primary outcome was postoperative leakage after surgery for duodenal trauma. The secondary outcome was efficacy of pyloric exclusion for postoperative leakage.

### 2.3. Statistical Analysis

We used the mean and standard deviation to express continuous variables. Categorical data are presented as proportions. Continuous data were compared using the Student’s *t*-test or Mann–Whitney U test as appropriate. Proportions were compared using the chi-square or Fisher’s exact tests as appropriate. *p* values < 0.05 were considered statistically significant. All statistical analyses were conducted using R language, version 4.1.2 (R foundation, Vienna, Austria). We used “autoReg”, “multipleROC”, “pROC”, “glmnet”, “tidyverse”, “ggplot2”, and “MASS” packages for data analysis and visualization.

We used fitting procedures that can yield better prediction accuracy and model interpretability as the number of observations was not much larger than the number of variables [10]. First, a stepwise multivariable logistic regression including forward and backward selections was used [11]. To adjust for confounding factors, variables with a univariable *p*-value < 0.20 were included in the multivariable logistic regression model. We used the Akaike information criterion (AIC) to choose the optimal model [11]. The AIC criterion is defined for a large class of models fit by maximum likelihood [11]. Second, we used a technique for shrinking the regression coefficients toward zero, which is known as the least absolute shrinkage and selection operator (LASSO) [10,12]. We used a 10-fold cross validation to select an optimal hyperparameter (lambda). In cross validation, optimal lambda was chosen as the most regularized model so that error was within one standard error of the minimum [10]. We input risk factors into the LASSO model such as age, gender, Charlson Comorbidity score, ISS, other abdominal injury, injury mechanism, OIS, injury site, pancreatic injury, type of duodenal operation, GCS, vasopressor use in emergency department (ED), time from injury to initial operation, operation time, and amount of transfusion.

We constructed a 2 by 2 contingency table according to specific cutoff values to calculate the accuracy, using parameters such as sensitivity, specificity, positive predictive value (PPV), and negative predictive value (NPV). A receiver operator characteristic (ROC) curve was also constructed to evaluate the diagnostic accuracy and calculate the area under the ROC curve (AUC). Youden’s index was used to determine the best cutoff value that maximizes the sensitivity and specificity [13].

## 3. Results

### 3.1. Patient Characteristics

During a 17-year period from January 2004 to December 2020, 179,887 trauma patients were admitted to the emergency room of our hospital. Of these, 73 patients (0.04%) were diagnosed with duodenal injuries through clinical work up. After implementation of exclusion criteria, a total of 49 consecutive patients with grade 2 and 3 traumatic duodenal injury, who underwent laparotomy were included. Clinical characteristics of patients with postoperative leakage and no leakage are summarized in Table 1. The incidence of postoperative leakage and mortality was 32.6% (16/49) and 18.4% (9/49), respectively. Mortality did not differ significantly (*p* = 0.219). Hospital stay was significantly longer in patients with leakage than in patients without leakage (26.2 ± 19.3 vs. 50.1 ± 31.5, *p* = 0.011). The time from injury to initial operation and operation time was significantly longer in patients with leakage than in patients without leakage (9.4 ± 5.1 vs. 31.4 ± 25.3; *p* = 0.003 and 180.0 ± 59.8 vs. 214.1 ± 42.9; *p* = 0.048, respectively). However, patients with no leakage received more transfusion of packed red blood cells (PRBC) within 24 h than patients with leakage (3.2 ± 4.9 vs. 1.2 ± 1.7, *p* = 0.048). In terms of other clinical variables, there was no significant difference.

### 3.2. Analysis of Risk Factors for Postoperative Duodenal Leakage

**Figure 1:**
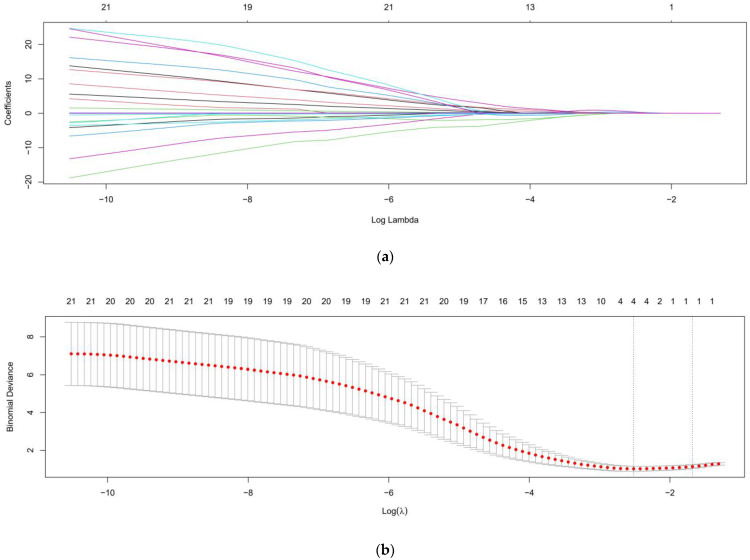
Clinical variables were selected using LASSO logistic regression model: (**a**) Shrinkage of coefficients by hyperparameter (λ); (**b**) Hyperparameter selection (λ) using cross validation. The dotted line indicates the value of the harmonic log (λ) when the error of model is minimized. In the LASSO logistic regression model, only one variable (time from injury to initial operation) was selected when log (λ) was −0.1772.

In a stepwise multivariable logistic regression including forward and backward selection, the backward model showed a more favorable AIC of 46.359, while the forward model showed an AIC of 50.594. Thus, we chose the backward model as a more appropriate model. In the backward stepwise model (Table 2), in the final analysis, time from injury to initial operation was the sole statistically significant risk factor (odds ratio [OR], 1.14; CI, 1.06 to 1.27; *p* = 0.006). In the forward model, time from injury to initial operation was also the sole significant risk factor (OR, 1.01; CI, 1.01 to 1.02; *p* < 0.001). The results of the LASSO logistic regression model are summarized in Figure 1. Figure 1a delineates shrinkage of coefficients by the hyperparameter (λ) and Figure 1b delineates accuracy of the model via cross validation. In cross validation, the optimal log (λ) was −0.1772. At this level, only one risk factor, the time from injury to initial operation, was selected and LASSO shrank the coefficient estimates of the other risk factors towards zero.

**Table 2:** Backward stepwise multivariable logistic regression model for postoperative leakage.

Variables	OR (Univariable)	OR (Multivariable)	OR (Final)
Duodenal 3rd portion injury (yes)	6.67 (1.75–33.41, *p* = 0.010)	1.71 (0.26–11.04, *p* = 0.564)	
GCS	1.49 (0.92–NA, *p* = 0.440)		
Time from injury to initial operation (hour)	1.14 (1.06–1.27, *p* = 0.006)	1.11 (1.04–1.25, *p* = 0.022)	1.14 (1.06–1.27, *p* = 0.006)
Operation time (min)	1.01 (1.00–1.03, *p* = 0.055)	1.01 (0.99–1.03, *p* = 0.262)	
PRBC transfusion within 24 h from admission (unit)	0.84 (0.62–1.02, *p* = 0.157)	0.88 (0.57–1.10, *p* = 0.419)	

GCS, Glasgow coma scale; PRBC, packed red blood cells; OR, odds ratio.

### 3.3. Performance of Selected Risk Factor

**Figure 2:**
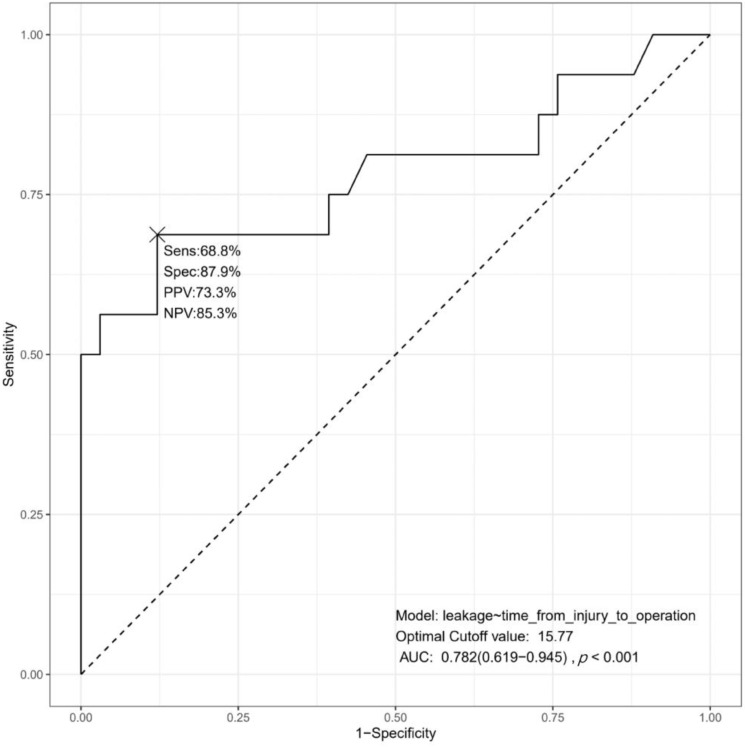
Diagnostic accuracy of time from injury to initial operation (hour) to detect the postoperative duodenal leakage. Optimal cutoff value is 15.77 h.

**Figure 3:**
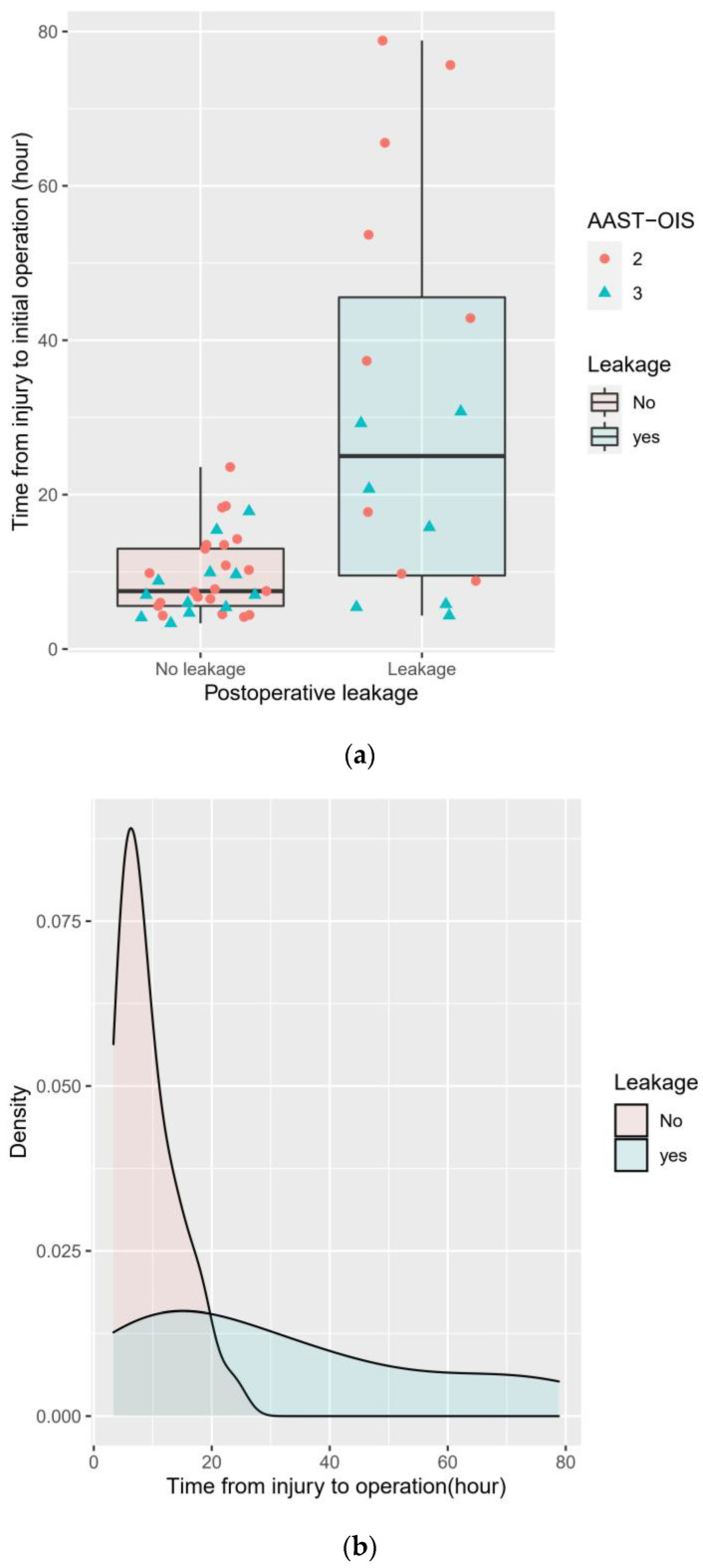

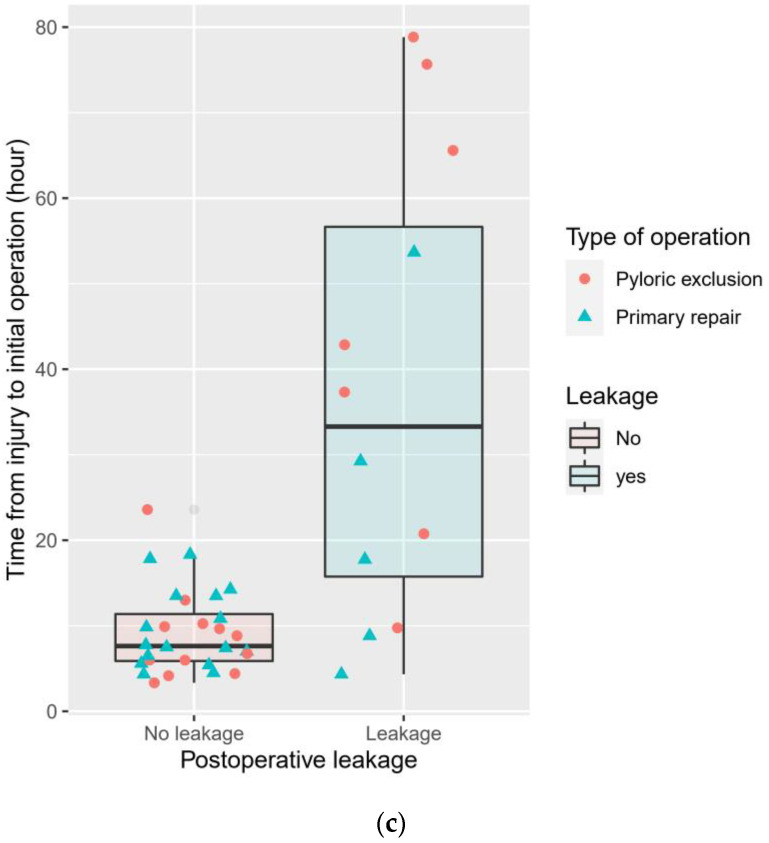
Box plot (**a**) and density plot (**b**) of time from injury to initial operation (hours) according to postoperative leakage following duodenal injury. Two types of surgery (primary closure alone and primary closure with pyloric exclusion) are delineated in box plot (**c**) after excluding other surgical procedures.

The ROC curve of time from injury to initial operation to predict postoperative duodenal leakage is shown in Figure 2. AUC was 0.782 (confidence interval [CI], 0.619 to 0.945; *p* < 0.001). The optimal cutoff value was 15.77 h, which has a 68.8% sensitivity, 87.9% specificity, 73.3% PPV, and 85.3% NPV. Figure 3a,b show the distribution of risk factors. In the no-leakage group, maximal time from injury to initial operation was 23.58 h. The predictive accuracy according to different thresholds is summarized in Table 3. At the threshold of 24 h, specificity and PPV were both 100%.

### 3.4. Efficacy of Pyloric Exclusion

We compared patients with primary repair alone (*n* = 21) and pyloric exclusion after primary repair (*n* = 19, Table 4). Patients with primary repair alone were significantly older than patients with pyloric exclusion after primary repair (52.8 ± 15.9 vs. 40.4 ± 16.6, *p* = 0.021), had higher Charlson Comorbidity scores (1.6 ± 1.5 vs. 0.6 ± 0.9, *p* = 0.017), and a higher number of injuries to the first portion of the duodenum (33.3% vs. 0%, *p* = 0.019). The operation time for pyloric exclusion was significantly longer than primary repair alone (156.2 ± 42.5 vs. 216.1 ± 47.5, *p* < 0.001). However, other clinical outcomes, including postoperative leakage, did not significantly differ. Figure 3c shows the distribution of the type of operation according to postoperative leakage and time from injury to initial operation in patients with primary repair alone and pyloric exclusion with primary repair.

## 4. Discussion

Traumatic duodenal injury is an extremely rare disease with limited evidence. We investigated the risk factors for postoperative duodenal leakage in patients with AAST-OIS 2 or 3 duodenal injury. Our study demonstrated that time from injury to initial operation may be the most significant and the sole risk factor to predict postoperative leakage. Other risk factors, such as AAST-OIS grade, operation time, GCS, vasopressor use, administration of transfusion, injury site of duodenum, and pyloric exclusion were not associated to postoperative leakage. Time from injury to initial operation showed good performance for prediction with an AUC of 0.782, 68.8% sensitivity, 87.9% specificity, 73.3% PPV, and 85.3% NPV at the optimal threshold of 15.77 h. Notably, at the threshold of 24 h, specificity and PPV were both 100%. We believe that our results could provide trauma surgeons meaningful insights. A further future prospective study is warranted but would be a major challenge in practice.

During our search of previous literature, we found that there was no randomized trial regarding duodenal trauma (Table 5). Due to extremely low incidence rates, conducting prospective studies is challenging. Moreover, the evidence regarding risk factors for postoperative duodenal leakage is substantially limited. In a recent retrospective study of 94 duodenal injuries in South Africa, Weale et al. [14] demonstrated that the incidence rate of postoperative leakage was statistically significant according to AAST-OIS (0% in grade 1, 1.6% in grade 2, and 66.7% in grade 3, *p* < 0.01). However, only three pyloric exclusions were performed by Weale et al. and 91 patients underwent primary repair alone; they only conducted a univariable analysis and not a multivariable analysis [14]. In a retrospective review of 222 duodenal injuries, including 88.3% of penetrating trauma injuries in a level 1 trauma center in the United States, Blocksom et al. [15] demonstrated that presence of an abdominal artery injury, ISS > 25, pancreatic injury, and low body temperature were significantly associated with infectious complications; however, they did not demonstrate the association with postoperative leakage. In a retrospective analysis including 125 penetrating duodenal traumas in the United States, Schroeppel et al. [16] demonstrated that patients with duodenal leakage were more likely to have a major vascular injury and a combined pancreatic injury. However, the authors found no differences in repair technique, location, or grade of injury [16]. Interestingly, the authors found that patients with duodenal leakage were more likely to have an extraluminal drain (90% vs. 45%, *p* = 0.008). In another retrospective review including 44 penetrating duodenal traumas in a level 1 trauma center in the United States, Ordonez et al. reported duodenal fistulas as the most common complication (33%) [17]. In our study, the incidence of postoperative duodenal leakage was 32.7% even in grade 2 or 3 injury. This is higher compared with older reviews, which range from 0% to 16.6% [1]. However, our cohort consisted of 87.8% blunt trauma injuries. The diagnosis of duodenal injury in blunt trauma is difficult and can be delayed [1]. This is due to the fact that an intact retroperitoneum may block the spillage of bowel content, which prevents panperitonitis. Indeed, retroperitonitis tends to be masked after injury because the bacterial count in the duodenum is low, and pancreatic bicarbonate can neutralize the gastric acid [5]. Delayed diagnosis can increase the contamination and inflammation of the operation site, which may contribute to decreased wound healing and increased risk of postoperative leakage. In our cohort, the incidence of vascular injury and pancreatic injury, which are known as significant risk factors [16], was low. Thus, our results suggest that time from injury to surgery may be the most important risk factor in a stable environment. The principle of parsimony might be suitable for this cohort. However, in a future study, the risk factor in patients with early intervention should be addressed because a duodenal fistula occurred in some patients in our study. Due to the small sample size, we could not address this issue.

Historically, several adjunctive operative techniques reduced the intraluminal pressure of the duodenum and blocked the influx of gastric content after repair of the injured duodenum. The goal of adjunctive operative variants is to prevent postoperative leakage and luminal narrowing. In 1968 and 1974, a trauma group from Los Angeles/University of Southern California described “Duodenal diverticulization”, which consisted of truncal vagotomy, antrectomy, gastrojejunostomy, T-tube drainage of the common bile duct, and a tube duodenostomy [18,30]. However, this procedure is time-consuming and often complicated due to possible sequelae of the vagotomy and the new hole in the normal tissue such as the antrum, duodenum, and common bile duct [1]. In our study, only one patient underwent duodenal diverticulization with damage control surgery due to instability and had no duodenal leakage. However, he died due to multiorgan failure. Pyloric exclusion with gastrojejunostomy was first described in 1907 [8]. In 1983, the Ben Taub trauma group from Houston, Texas conducted a retrospective study including 128 duodenal traumas and reported decreased duodenal fistula and fewer complications after pyloric exclusion [20]. However, recent literature has reported a trend toward a less invasive operative procedure. In a retrospective study using NTDB, Dubose et al. reported that pyloric exclusion was associated with longer hospital stays and had no mortality benefit [25]. Notably, in a retrospective study, Aiolfi et al. used NTDB and found a progressive trend toward less invasive procedures and improved mortality in the late period (2002–2006 vs. 2007–2014) [3]. Ferrada et al. [27], in a recent international retrospective study involving 13 centers with 372 duodenal injuries (79% penetrating injuries), reported that primary repair alone is amenable in the majority of patients (80%), even with high grade duodenal injury (grades 4 and 5). Notably, they reported that duodenal leakage was statistically significantly lower in patients with primary repair alone [27]. In an older study, the pyloric exclusion opened up between 14 and 21 postoperative days in 94% patients [20]. Thus, pyloric exclusion can protect the duodenal repair by blocking the influx of gastric content only in the first two or three postoperative weeks. Thus, the impact of pyloric exclusion may be minimal, although it is a more complex procedure requiring a longer operation time than primary repair alone. Even in our study, we found that the pyloric exclusion technique did not provide any superior outcome compared with primary repair alone. However, to provide nutritional support, a Witzel feeding jejunostomy may be considered as an alternative for patients with a fistula or high-risk patients who have the potential of fistula; high risk may be defined as delayed operation or delayed diagnosis, as per our results. In our retrospective chart review of patients, the reason for delayed surgery was no evidence of duodenal perforation or severe duodenal injury on the clinical and radiologic diagnosis. Fang et al. [22] reported 18 delayed duodenal injuries that were not diagnosed within the first 24 h. They reported that the reasons for delayed diagnosis were injuries not found during the first operation (*n* = 6), delay in seeking medical help (*n* = 6), conservative treatment at local hospital (*n* = 5), and delay in their hospital (*n* = 1) [22]. However, in our study, the exact reason for misdiagnosis or delayed perforation remains unknown. We may, therefore, have to focus on operative strategies, such as feeding jejunostomy or postoperative monitoring strategies, when we encounter duodenal perforation with delayed diagnosis. In our study, damage control surgery was rare (three patients) and showed no postoperative leakage. Thus, the impact of damage control surgery is unclear and future study regarding this issue is needed.

Our study had several limitations. First, the limited number of patients and retrospective nature of the study can induce substantial bias, such as selection bias or survival bias. A future prospective study with a large cohort is warranted; however, this trial would be challenging to undertake due to the low incidence of duodenal trauma. The limited number of observations may induce increased variance, which may then increase instability of regression and induce overfitting [10]. Moreover, the type of surgery, such as pyloric exclusion and primary repair alone, was substantially heterogeneous. The extent of surgery may indicate a more severe duodenal injury and a higher risk of postoperative leakage. Thus, our findings may have low strength of evidence due to the substantial heterogeneity of the included patients and small sample size, which may induce substantial bias. To overcome this issue, we used a stepwise selection model and LASSO regression model. Furthermore, we compared the pyloric exclusion group with the primary repair alone group to investigate a secondary outcome. Second, we constructed a cohort with grade 2 and grade 3 duodenal injury. This categorization was arbitrary, being based on our researcher’s decision. Some literature defines high grade injury as grade 3 or more; however, postoperative leakage occurred in 26% (8/30) patients with grade 2 duodenal injury. This implies that it is insufficient to determine the injury severity only by the circumference of the perforated bowel and that a hidden risk factor may exist. Based on our results, the time from injury to initial operation is a more important risk factor than the AAST-OIS injury grade. Third, the surgeons’ skill level and experience varied. This may interfere with the consistency of the quality of operations and decisions in the surgical field. The overall management of duodenal trauma or surgical technique may have evolved in our institution. However, there was no significant difference when we divided the period: 42.8% (9/21) patients showed duodenal fistula in the early period (2004–2010) and 25.0% (7/28) patients in the late period (2011–2020) (*P* = 0.312). However, the learning curve at the hospital level may improve and future study is needed. Fourth, we excluded the patients who died by postoperative day 7. This exclusion criterion was arbitrary. Two patients who were excluded died on postoperative day 1. In general, postoperative leakage does not occur within a short period. Another criterion, such as postoperative day 3, could be used in the future. Finally, decisions by surgeons to perform pyloric exclusion were arbitrary and heuristic. As there is a lack of clear guidelines, a surgeon’s decision depended on intuitive assessment or the gestalt of the surgeon. More specific guidelines are thus warranted.

## 5. Conclusions

Time from injury to initial operation may be the sole significant risk factor for postoperative duodenal leakage. Pyloric exclusion may not be able to prevent postoperative leakage. In patients with a delayed operation, careful consideration regarding surgical strategy and close observations are needed.

## Figures and Tables

**Table 1:** Difference in clinical characteristics of patients with postoperative leakage and with no leakage.

	Postoperative Leakage		
Variables	No Leakage (*n* = 33)	Leakage (*n* = 16)	*p*
Age (year, Mean ± SD)	47.8 ± 17.9	49.1 ± 15.4	0.804
Gender			
F	8 (24.2%)	4 (25%)	1.000
M	25 (75.8%)	12 (75%)	
Hospital stay (day, Mean ± SD)	26.2 ± 19.3	50.1 ± 31.5	0.011
Charlson Comorbidity index (Mean ± SD)	1.2 ± 1.4	1.1 ± 1.4	0.728
ISS (Mean ± SD)	12.6 ± 7.6	10.2 ± 4.8	0.246
Injury mechanism			
Blunt	28 (84.8%)	15 (93.8%)	0.670
Penetrating	5 (15.2%)	1 (6.2%)	
Duodenal organ injury scale			
2	21 (63.6%)	9 (56.2%)	0.853
3	12 (36.4%)	7 (43.8%)	
Injured site of duodenum			
1st portion	9 (27.3%)	2 (12.5%)	0.425
2nd portion	10 (30.3%)	2 (12.5%)	0.315
3rd portion	13 (39.4%)	13 (81.2%)	0.014
4th portion	2 (6.1%)	1 (6.2%)	1.000
Other abdominal organ injury	16 (48.5%)	9 (56.2%)	0.837
Accompanied pancreatic injury	5 (15.2%)	2 (12.5%)	1.000
Accompanied pancreatic injury (≥grade 3)	1 (3%)	0 (0%)	1.000
Other hollow viscus organ injury	6 (18.2%)	3 (18.8%)	1.000
Major abdominal vascular injury	2 (6.1%)	1 (6.2%)	1.000
Type of duodenal surgery			
Antrectomy with gastrojejunostomy	2 (6.1%)	1 (6.2%)	0.480
Duodenal diverticulization	1 (3%)	0 (0%)	
Duodenojejunostomy	0 (0%)	1 (6.2%)	
Duodenojejunostomy + PE	1 (3%)	0 (0%)	
Duodenal primary repair + PE	12 (36.4%)	7 (43.8%)	
Duodenal primary repair alone	16 (48.5%)	5 (31.2%)	
Duodenal resection with anastomosis + PE	1 (3%)	2 (12.5%)	
Damage control surgery	3 (9.1%)	0 (0%)	0.542
Mortality	4 (12.1%)	5 (31.2%)	0.219
GCS (Mean ± SD)	14.2 ± 2.7	14.9 ± 0.2	0.110
Vasopressor use in ED	5 (15.2%)	3 (18.8%)	1.000
Time from injury to initial operation (hour, Mean ± SD)	9.4 ± 5.1	31.4 ± 25.3	0.003
Operation time (min, Mean ± SD)	180.0 ± 59.8	214.1 ± 42.9	0.048
Other abdominal complications	5 (15.2%)	6 (37.5%)	0.164
Systemic complication	4 (12.1%)	4 (25%)	0.464
Dindo classification			
0	22 (66.7%)	4 (25%)	<0.001
1	7 (21.2%)	0 (0%)	
2	0 (0%)	6 (37.5%)	
3a	0 (0%)	1 (6.2%)	
3b	1 (3%)	0 (0%)	
4a	1 (3%)	0 (0%)	
4b	1 (3%)	0 (0%)	
5	1 (3%)	5 (31.2%)	
PRBC transfusion within 24 h from admission (unit, Mean ± SD)	3.2 ± 4.9	1.2 ± 1.7	0.048
FFP transfusion within 24 h from admission (unit, Mean ± SD)	1.7 ± 3.2	1.1 ± 1.8	0.427
PLT transfusion within 24 h from admission (unit, Mean ± SD)	1.3 ± 3.3	1.2 ± 3.4	0.935

ISS, injury severity score; PE, pyloric exclusion with gastrojejunostomy; GCS, Glasgow coma scale; ED, emergency department; PRBC, packed red blood cells; FFP, fresh frozen plasma; PLT, platelet; SD, standard deviation.

**Table 3:** Predictive accuracy for postoperative leakage according to different thresholds (time from injury to initial operation).

Threshold	Sensitivity	Specificity	PPV	NPV
6 h	0.81	0.30	0.36	0.77
12 h	0.69	0.73	0.55	0.83
15.77 h (optimal)	0.69	0.88	0.73	0.85
24 h	0.50	1.00	1.00	0.80

PPV, positive predictive value; NPV, negative predictive value.

**Table 4:** Comparison between two types of surgery (primary repair alone vs. primary repair + pyloric exclusion).

	Operation Type		*p*
Variables	Primary Repair Alone (*n* = 21)	Primary Repair + Pyloric Exclusion (*n* = 19)	
Age	52.8 ± 15.9	40.4 ± 16.6	0.021
Gender			
F	5 (23.8%)	6 (31.6%)	0.845
M	16 (76.2%)	13 (68.4%)	
Hospital stay (day, Mean ± SD)	29.6 ± 23.1	36.3 ± 22.0	0.351
Charlson Comorbidity index (Mean ± SD)	1.6 ± 1.5	0.6 ± 0.9	0.017
ISS (Mean ± SD)	12.2 ± 7.2	11.3 ± 7.3	0.689
Injury mechanism			
Blunt	17 (81%)	18 (94.7%)	0.402
Penetrating	4 (19%)	1 (5.3%)	
Duodenal organ injury scale			
2	16 (76.2%)	13 (68.4%)	0.845
3	5 (23.8%)	6 (31.6%)	
Injured site of duodenum			
1st portion	7 (33.3%)	0 (0%)	0.019
2nd portion	7 (33.3%)	5 (26.3%)	0.890
3rd portion	9 (42.9%)	13 (68.4%)	0.192
4th portion	1 (4.8%)	1 (5.3%)	1.000
Other abdominal organ injury	10 (47.6%)	9 (47.4%)	1.000
Accompanied pancreatic injury	1 (4.8%)	1 (5.3%)	1.000
Other hollow viscus organ injury	5 (23.8%)	3 (15.8%)	0.812
Major abdominal vascular injury	0 (0%)	1 (5.3%)	0.960
Postoperative leakage	5 (23.8%)	7 (36.8%)	0.580
Damage control surgery	1 (4.8%)	0 (0%)	1.000
Mortality	3 (14.3%)	2 (10.5%)	1.000
GCS (Mean ± SD)	14.5 ± 2.0	14.4 ± 2.8	0.837
Vasopressor use in ED	3 (14.3%)	1 (5.3%)	0.673
Time from injury to initial operation (hour, Mean ± SD)	12.8 ± 11.2	23.0 ± 25.0	0.114
Operation time (min, Mean ± SD)	156.2 ± 42.5	216.1 ± 47.5	<0.001
Other abdominal complication	3 (14.3%)	6 (31.6%)	0.353
Systemic complication	4 (19%)	0 (0%)	0.140
Dindo classification			
0	14 (66.7%)	9 (47.4%)	1.000
1	3 (14.3%)	3 (15.8%)	
2	0 (0%)	4 (21.1%)	
3a	1 (4.8%)	0 (0%)	
3b	0 (0%)	1 (5.3%)	
4a	1 (4.8%)	0 (0%)	
4b	0 (0%)	0 (0%)	
5	2 (9.5%)	2 (10.5%)	
PRBC transfusion within 24 h from admission (unit, Mean ± SD)	2.3 ± 3.8	1.4 ± 2.6	0.361
FFP transfusion within 24 h from admission (unit, Mean ± SD)	0.9 ± 1.5	1.0 ± 2.5	0.887
PLT transfusion within 24 h from admission (unit, Mean ± SD)	1.6 ± 3.5	1.1 ± 3.2	0.595

ISS, injury severity score; PE, pyloric exclusion with gastrojejunostomy; GCS, Glasgow coma scale; ED, emergency department; PRBC, packed red blood cell; FFP, fresh frozen plasma; PLT, platelet; SD, standard deviation.

**Table 5:** Literature review of duodenal trauma.

Author	Year	Study Design	Location	Inclusion	Number of Patients with Duodenal Trauma	Incidence
Berne et al. [18]	1968	Retrospective, single center (six years)	USA	Severe duodenal trauma	16	Not reported
Lucas et al. [4]	1975	Retrospective, single center (1960–1974)	USA	Blunt duodenal trauma	36	Not reported
Vaughan et al. [19]	1977	Retrospective, single center (1969–1976)	USA	Duodenal trauma	175	Not reported
Martin et al. [20]	1983	Retrospective, single center (1969–1980)	USA	Duodenal trauma	313	Not reported
Ivatury et al. [21]	1985	Retrospective, single center (1972–1984)	USA	Penetrating duodenal trauma	100	Not reported
Fang et al. [22]	1999	Retrospective, single center (1986–1995)	Taiwan	Delayed diagnosed duodenal injury	18	Not reported
Jansen et al. [23]	2002	Retrospective, single center (1997–1999)	South Africa	Surgically identified duodenal injury	30	Not reported
Blocksom et al. [15]	2004	Retrospective, single center (1980–2002)	USA	Duodenal trauma with laparotomy	222	Not reported
Huerta et al. [24]	2005	Retrospective, single center (1996–2003)	USA	Duodenal trauma	52	0.49% (52/10584)
Dubose et al. [25]	2008	Retrospective, NTDB (five years)	USA	AAST ≥ 3 duodenal trauma with primary repair or pyloric exclusion	147	0.015% (147/952242)
Ordonez et al. [17]	2013	Retrospective, single center (2003–2012)	USA	Penetrating duodenal trauma	44	Not reported
Schroeppel et al. [16]	2015	Retrospective, single center (1996–2014)	USA	Penetrating duodenal trauma	212	Not reported
Phillips et al. [26]	2017	Retrospective, NTDB (2010–2014)	USA	Penetrating duodenal trauma	879	0.09% (879/4030635)
Ferrada et al. [27]	2018	Retrospective, 13 centers (2007–2016)	USA, Brazil, Panama	Duodenal trauma requiring surgery	372	Not reported
Aiolfi et al. [3]	2019	Retrospective, NTDB (2002-2014)	USA	AAST ≥ 3 duodenal trauma	2163	Not reported
Weale et al. [14]	2019	Retrospective, single center (2012–2016)	South Africa	Duodenal trauma requiring surgery	94	Not reported
Turan et al. [28]	2020	Retrospective, single center (2011–2018)	Turkey	Penetrating duodenal trauma with primary repair	26	Not reported
Butano et al. [29]	2021	Retrospective, single center (2013–2020)	USA	Duodenal trauma	23	Not reported

NTDB, National Trauma Database; AAST, American Association for the Surgery for Trauma.

## Data Availability

Not applicable.
